# Cloning and Characterization of a Norbelladine 4′-*O-*Methyltransferase Involved in the Biosynthesis of the Alzheimer’s Drug Galanthamine in *Narcissus* sp. *aff. pseudonarcissus*

**DOI:** 10.1371/journal.pone.0103223

**Published:** 2014-07-25

**Authors:** Matthew B. Kilgore, Megan M. Augustin, Courtney M. Starks, Mark O’Neil-Johnson, Gregory D. May, John A. Crow, Toni M. Kutchan

**Affiliations:** 1 Donald Danforth Plant Science Center, St. Louis, Missouri, United States of America; 2 Sequoia Sciences, St Louis, Missouri, United States of America; 3 National Center for Genome Resources, Santa Fe, New Mexico, United States of America; University of New South Wales, Australia

## Abstract

Galanthamine is an Amaryllidaceae alkaloid used to treat the symptoms of Alzheimer’s disease. This compound is primarily isolated from daffodil *(Narcissus* spp.*)*, snowdrop (*Galanthus* spp.), and summer snowflake (*Leucojum aestivum)*. Despite its importance as a medicine, no genes involved in the biosynthetic pathway of galanthamine have been identified. This absence of genetic information on biosynthetic pathways is a limiting factor in the development of synthetic biology platforms for many important botanical medicines. The paucity of information is largely due to the limitations of traditional methods for finding biochemical pathway enzymes and genes in non-model organisms. A new bioinformatic approach using several recent technological improvements was applied to search for genes in the proposed galanthamine biosynthetic pathway, first targeting methyltransferases due to strong signature amino acid sequences in the proteins. Using Illumina sequencing, a *de novo* transcriptome assembly was constructed for daffodil. BLAST was used to identify sequences that contain signatures for plant *O*-methyltransferases in this transcriptome. The program HAYSTACK was then used to identify methyltransferases that fit a model for galanthamine biosynthesis in leaf, bulb and inflorescence tissues. One candidate gene for the methylation of norbelladine to 4′-*O*-methylnorbelladine in the proposed galanthamine biosynthetic pathway was identified. This methyltransferase cDNA was expressed in *E. coli* and the protein purified by affinity chromatography. The resulting protein was found to be a norbelladine 4′-*O*-methyltransferase (*Np*N4OMT) of the proposed galanthamine biosynthetic pathway.

## Introduction

Amaryllidaceae alkaloids are a group of alkaloids with many documented biological activities. This makes them valuable potential medicines several examples are the anti-cancer compounds hemanthamine and lycorine and the anti-viral compound pancratistatin [1]–[3]. One example of an Amaryllidaceae alkaloid already used medically to treat Alzheimer’s disease is galanthamine. Galanthamine is an alkaloid discovered in 1953 that is produced by members of the Amaryllidaceae family [4]. It reduces the symptoms of Alzheimer’s disease through acetylcholine esterase inhibition and nicotinic receptor binding. These activities are thought to compensate for reduced acetylcholine sensitivity in Alzheimer’s disease by increasing acetylcholine levels and perhaps increasing acetylcholine sensitivity [5], [6]. Until now, no committed biosynthetic genes have been identified [7], [8]. Limited enzyme kinetic characterization has been done on plant protein extracts enriched for the norbelladine 4′-*O*-methyltransferase (N4OMT) of *Nerine bowdenii*, but the underlying gene was never identified [9].

**Figure 1:**
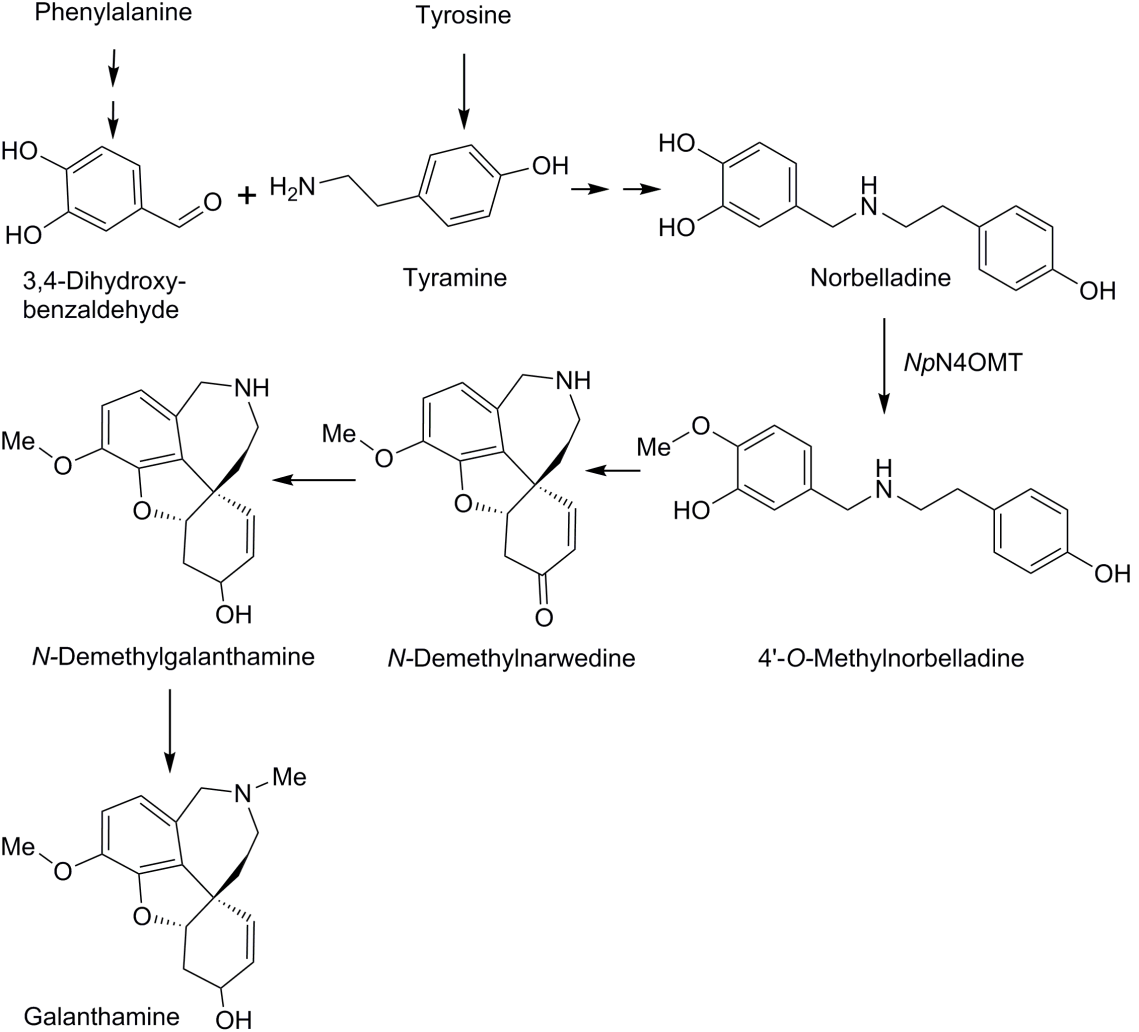
Proposed biosynthetic pathway for galanthamine. 3,4-Dihydroxybenzaldehyde derived from phenylalanine and tyramine derived from tyrosine are condensed to form norbelladine. Norbelladine is methylated by *Np*N4OMT to 4′-*O-*methylnorbelladine. 4′-*O-*Methylnorbelladine is oxidized to *N-*demethylnarwedine. *N-*demethylnarwedine is then reduced to *N-*demethylgalanthamine. In the last step, *N-*demethylgalanthamine is methylated to galanthamine.

The putative galanthamine biosynthetic pathway has been studied in detail and intermediates in the pathway have been determined. This knowledge is based on radiolabeling experiments. Work on other Amaryllidaceae alkaloids including lycorine and hemanthamine studying steps prior to 4′-*O*-methylnorbelladine can be applied to galanthamine biosynthesis because 4′-*O*-methylnorbelladine is a universal substrate for these alkaloids [10]. The proposed pathway starts with the amino acids phenylalanine and tyrosine [11]. In *Narcissus incomparabilis* phenylalanine was established as a precursor that contributes the catechol portion of norbelladine. This was done using radiolabeling experiments to trace incorporation of [3-^14^C]phenylalanine into lycorine and degradation experiments on the resulting lycorine to determine the location of the ^14^C label [12]. Similar experiments with phenylalanine were performed in *Nerine browdenii* monitoring hemanthamine incorporation [13]. As a follow up radiolabeling experiments were used to determine that phenylalanine probably proceeds sequentially through the intermediates *trans*-cinnamic acid, *p*-hydroxycinnamic acid and 3,4-dihydroxycinnamic acid or *p*-hydroxybenzaldehyde before conversion into 3,4-dihydroxybenzaldehyde [14]. Tyrosine has been established as a precursor of galanthamine that in contrast to phenylalanine contributes only to the non-catechol half of the norbelladine intermediate. This was done by observing [2-^14^C]tyrosine incorporation into galanthamine and degradation experiments of galanthamine [11]. Tyrosine decarboxylase converts tyrosine into tyramine and is well characterized in other plant families [15]. 3,4-Dihydroxybenzaldehyde and tyramine condense into a Schiff-base and are reduced to form the first alkaloid in the proposed pathway, norbelladine. Norbelladine has been documented to incorporate into galanthamine and all major Amaryllidaceae alkaloid types in ^14^C radiolabeling studies [11], [16]–[18]. 4′-*O*-methylnorbelladine is then formed by *O*-methylation of norbelladine [11]. A phenol-coupling reaction, followed by spontaneous oxide bridge formation, creates *N*-demethylnarwedine, which is then reduced and *N*-methylated to yield galanthamine (Figure 1) [10]. In one study, Barton et al. fed *O*-methyl[1-^14^C]norbelladine to flower stalks of King Alfred daffodils, but it was not incorporated into galanthamine. The authors concluded that the intermediate in the pathway must be 4′-*O*-methyl-*N*-methylnorbelladine despite low incorporation of this compound when the equivalent experiment was conducted with 4′-O-methyl-[N-methyl-^14^C]norbelladine [11]. A recent revision of the proposed pathway by Eichhorn et al. contradicted this conclusion and placed the *N*-methylation step at the end of the proposed pathway instead of before the phenol-coupling reaction. In that study, [OC^3^H_3_]4′-*O*-methylnorbelladine was applied to ovary walls of *Leucojum aestivum.* Incorporation into products indicated that the pathway produced *N*-demethylated intermediates up until the penultimate step to galanthamine. *N*-methylation was proposed as the final step of biosynthesis [10].

The discovery of genes involved in metabolism is essential to metabolic engineering and synthetic biology. The elucidation of plant biochemical pathways can take decades. In fact, the biosynthesis of morphine, an important opiate analgesic, is still not completely elucidated at the gene level, even though the first enzyme specific to morphine biosynthesis was discovered more than 20 years ago in 1993 [19]. Reports on the enzymatic activities of poppy extracts to describe the morphine biosynthetic pathway go even farther back to 1971 [20]. After more than 40 years of enzymology and reverse genetics, the morphine biosynthetic pathway is still incomplete at the gene level. Traditionally, plant biochemical pathway enzymes have been identified either directly by purification from plant extracts or indirectly by examining enriched cDNA libraries and functionally expressing clones [9], [21]–[27]. To reduce pathway discovery from a 20+ year process to a more reasonable time frame, new methods must be developed and embraced. The previous work on galanthamine biosynthesis makes the prediction of enzyme classes involved in the proposed pathway possible, thereby rendering the galanthamine pathway a suitable system for development of an omic methodology for biochemical pathway discovery.

In this study, using galanthamine biosynthesis as proof-of-concept, a novel workflow is presented to streamline the identification of biosynthetic pathway genes. A *de novo* transcriptome is created for *Narcissus* sp. *aff. pseudonarcissus* using illumina sequencing. HAYSTACK, a program that utilizes the Pearson correlation, is used to find genes that co-express with galanthamine accumulation in this transcriptome. This set of candidates is interrogated for homologs to methyltransferases. An OMT that converts norbelladine to 4′-*O*-methylnorbelladine (*Np*N4OMT) in the proposed biosynthesis of galanthamine is identified in this manner and characterized.

## Materials and Methods

### Plant tissue and chemicals

Daffodil plants were collected from an outdoor plot in St. Louis, MO, with the GPS coordinates (38.659305, −90.410203), during peak flowering and separated into leaf, bulb and inflorescence tissues. Inflorescence is considered all tissues above the spathe. The plants were collected with the permission of the corresponding author who is the owner of the private property. No endangered species were involved in this collection.

Formic acid, potassium phosphate monobasic, potassium phosphate dibasic, tris(hydroxymethyl)aminomethane, glycerol, sodium acetate, sodium chloride, tetramethylethylenediamine, calcium chloride, magnesium chloride and β-mercaptoethanol were obtained from Acros Organics. Glycine, papaverine hydrochloride, *S*-adenosyl methionine (AdoMet), cobalt chloride, zinc chloride and manganese chloride were obtained from Fisher Scientific. Other chemicals include acetonitrile, JT Baker; InstaPAGE, IBI Scientific; ethanol 200 proof, KOPTEC; Bradford reagent, Bio-Rad; *S*-adenosyl-L-homocysteine, Sigma-Aldrich; deoxynucleotide triphosphates (dNTPs), New England BioLabs, Inc. (NEB); and isopropyl β-D-1-thiogalactopyranoside (IPTG), Gold Biotechnology. The norbelladine *N*-methylnorbelladine, 4′-*O*-methyl-*N*-methylnorbelladine and 4′-*O-*methylnorbelladine were synthesized previously [10]. NotI, NdeI, T4 DNA ligase, Taq DNA Polymerase and Phusion High-Fidelity DNA Polymerase enzymes were from NEB. M-MLV reverse transcriptase and RNaseOUT were obtained from Invitrogen.

### Alkaloid extraction and quantification

Daffodil leaf, bulb and inflorescence tissues were extracted by grinding tissue with a mortar and pestle cooled with liquid nitrogen. Each ground sample was split into three technical replicates. Two volumes of 70% ethanol were added followed by vortexing 5 min and centrifuging at 14,000×g for 10 min. The supernatant was filtered through a 0.2 µm low protein binding hydrophilic LCR (PTFE, millex-LG) membrane. For galanthamine quantitation, samples were diluted 1000 fold. Liquid chromatography samples were injected (10 µl) onto an LC-20AD (Shimadzu) with a Waters Nova Pak C-18 (300×3.9 mm 4 µm) column coupled to a 4000 QTRAP (AB Sciex Instruments) for MS/MS analysis. The gradient program had a flow rate of 0.8 ml/min; solvent A was 0.1% formic acid in H_2_O and solvent B was 0.1% formic acid in acetonitrile. At the beginning of the program, solvent B was held at 15% for 2 min, followed by a linear gradient to 43% B at 15 min, 90% B at 15.1 min, 90% B at 20 min, 15% B at 21 min and 15% B at 26 min. A Turbo Ion Spray ionization source temperature of 500°C was used with low resolution for Q1 and Q3. All multiple reaction monitoring (MRM) scans were performed in positive ion mode. The ion fragment used for quantitation of galanthamine was 288.00 [M+H]^+^/213.00 [M-OH-C_3_H_7_N]^+•^ *m/z*. Galanthamine was identified by comparison of retention time and fragmentation pattern to authentic galanthamine standard. The Analyst 1.5 software was used to quantitate galanthamine using a comparison of peak area of the unknown to authentic galanthamine.

### Illumina sequencing and transcriptome assembly

The transcriptome was generated via data cleaning, short read assembly, final assembly, and post processing steps. A modified TRIzol RNA isolation method found as protocol number 13 in Johnson et al. was used to obtain RNA for cDNA library preparation [28]. Illumina RNA-Seq was used to generate 100 base pair paired-end reads from the cDNA library. The resulting data was monitored for overrepresented reads. Having found no such reads, adaptor sequences and sections of the standard phi X genome were identified and removed. Reads were then trimmed for quality using the FASTX toolkit version 0.0.8 with a Q value cutoff of 10 as is default for PHRAP [29].

Reads were assembled in the following manner. ABySS was used to run multiple assemblies of the reads with a range of kmers 24≤k≤54. The resulting assemblies were assembled into scaffolds using ABySS scaffolder [30]. Gaps in the sequences were resolved using GapCloser from the SOAPdenovo suit [31]. A final assembly was conducted on the resulting synthetic ESTs using Mira in EST assembly mode [32]. All sequences with over 98% identity were considered redundant and removed using CD-Hit [33]. The resulting contigs >100 base pairs long were included in the final assembly. Protein products for these contigs were predicted using ESTScan [34], [35]; all peptides over 30 amino acids were reported. Burrows-Wheeler Aligner was used to align the original reads to the assembled transcriptome to generate relative expression data for the contigs in leaf, bulb and inflorescence tissues [36]. The daffodil assembly and the raw read data can be found at the MedPlant RNA Seq Database, http://www.medplantrnaseq.org. Anomalies in the number of reads per contig and abnormally long or short contigs were manually curated. To normalize for read depth, each expression value for each contig was divided by the total reads for the respective tissue and multiplied by 1 million.

### Candidate gene identification

Relative expression data was compared to the levels of galanthamine in daffodil tissues using HAYSTACK with a background cutoff of 1, correlation cutoff 0.8, fold cutoff 4 and p-value 0.05 [37]. Using BLASTP, a list of known methyltransferases were queried against the daffodil transcriptome peptide list with an E-value of e^−9^ to identify methyltransferase homologs [38]. Accession numbers from NCBI for these methyltransferases are presented in Table S1. Overlap between the methyltransferase homologs and contigs that pass the HAYSTACK criteria were considered candidate genes. The candidate daffodil norbelladine 4′-OMT has the designation medp_9narc_20101112|62361 in the contigs.fa file in the Narcissus_spp.tar file on http://www.medplantrnaseq.org.

### Phylogenetic tree

Sequences found in Table S2 were aligned using MUSCLE in the MEGA 5.2 software with default parameters [39]. For the phylogeny, this alignment was provided as input into the Maximum-Likelihood algorithm also found in MEGA 5.2. Default parameters were used except the Gaps/Missing Data treatment was set to partial deletion.

### PCR and Cloning

The 5′ and 3′ ends of the *Np*N4OMT sequence were completed using Rapid Amplification of cDNA Ends (RACE) with the Invitrogen RACE kit. For gene specific primers (GSP) see Table S3. The same PCR program was used for both 5′ and 3′RACE. This applies to both cycles of nested PCR as well. The PCR program parameters were 30 s 98°C 1 cycle; 10 s 98°C, 30 s 60°C, 1 min 72°C 30 cycles; 5 min 72°C 1 cycle. The outer-primer PCR was a mixture of 4.6 ng/µl RACE ready bulb cDNA, 0.3 mM dNTPs, 0.3 µM GSP primer, 0.9 µM kit provided RACE primer, 1 U NEB Phusion High-Fidelity DNA Polymerase and Invitrogen recommended quantity of buffer in a 50 µl reaction. The inner-primer PCR used the product of the outer-primer PCR as template with 0.2 µM of the inner RACE GSP and Invitrogen primers and 0.2 mM dNTPs.

Amplification of the *Np*N4OMT open reading frame was performed with 5.1 ng/µl daffodil bulb oligo(dT) primed cDNA, 0.4 mM dNTPs, 0.4 µM each forward and reverse outer-primer, 1 UNEB Phusion High-Fidelity DNA Polymerase and recommended buffer in a 50 µl reaction. With the following PCR program parameters: 30 s 98°C 1 cycle; 10 s 98°C, 30 s 52°C, 1 min 72°C for 30 cycles; 5 min 72°C 1 cycle. The inner-primer PCR used 1 µl of the outer-primer PCR product and used the inner-primers in Table S3. The same PCR time program was used except the annealing temperature was increased to 53°C.

*Np*N4OMT was cloned into the pET28a vector with the NotI and NdeI restriction sites that were added to the 5′ and 3′ ends of the open reading frame using the inner PCR primers. PCR product and pET28a were digested with NotI and NdeI enzymes, followed by gel purification and ligation with the T4 DNA ligase. The resulting construct was transformed into *E. coli* DH5α cells and screened on Luria-Bertani agar plates with 50 µg/ml kanamycin. Resulting colonies were screened by colony PCR with T7 sequencing and T7 terminator primers and Taq DNA Polymerase. The following cycle program was used: 3 min 94°C 1 cycle; 30 s 94°C, 30 s 52°C, 2 min 72°C 30 cycles; 7 min 72°C 1 cycle. Plasmid minipreps were obtained using the QIAGEN QIAprep Spin Miniprep Kit. After Sanger sequencing of constructs (Genewiz), the desired plasmids were transformed into *E. coli* BL21(DE3) Codon Plus RIL competent cells. The sequences of the resulting five variants have the following accession numbers KJ584561(NpN4OMT1), KJ584562(*Np*N4OMT2), KJ584563(*Np*N4OMT3), KJ584564(*Np*N4OMT4) and KJ584565(*Np*N4OMT5).

### Protein purification

Recombinant protein production in 1 L of *E. coli* and purification with TALON resin followed the protocol found in [40]. No proteases were added to the protein extract, and desalting was performed with PD-10 columns from GE Healthcare. Protein quantity was determined according to Bradford; purity was monitored by SDS-PAGE. The *E. coli* cell line containing the hexahistidine-tagged methylthioadenosine/*S*-adenosylhomocysteine nucleosidase (Pfs) construct from Choi-Rhee and Cronan’s work was used to purify Pfs protein [41].

### Screening enzyme assays

Enzyme assays for initial testing of *Np*N4OMT1 contained 10 µg of pure protein with 200 µM AdoMet, 100 µM norbelladine and 30 mM potassium phosphate buffer pH 8.0 in 100 µl. The assays were incubated for 2 hr at 30°C. The vector control was an *E. coli* extract purified with TALON in the same way as the methyltransferase protein. For the vector control assay, an equal volume of the pure vector control extract was substituted for the *Np*N4OMT1 protein in the enzyme assay. These assays were quenched by adjusting the pH to 9.5 with two volumes of sodium bicarbonate and extracted with two volumes ethyl acetate two times. After drying, the extracts were re-suspended in the initial mobile phase of the HPLC program. The HPLC separation of the assays was performed using a phenomenex Luna C8(2) 5 µm 250×4.6 mm column with solvent A (0.1% formic acid in H_2_O) and solvent B (acetonitrile). The program started with 10% solvent B and a flow rate of 0.8 ml/min, a linear gradient began at 2 min to 30% at 15 min, 90% at 15.1 min, 90% at 20 min, 10% at 21 min and 10% at 28 min. Injection volume was 20 µl using a Waters Autosampler. Waters UV detector was set to 277 nm.

### Kinetic characterization

After optimization of the assay, the buffer was changed to 100 µM glycine at pH 8.8, with 5 mM of MgCl_2_ added and the temperature was increased to 37°C in 100 µl total reaction volume. When performing kinetic assays, the *E. coli* enzyme Pfs was added to break down SAH and prevent product inhibition. Product formation in kinetic experiments was quantified by comparing product peak area to a standard curve of the expected product or equivalent. Papaverine was used as an internal standard.

With the same solvent system as for screening enzyme assays, the HPLC program started with 20% B and a flow rate of 0.8 ml/min, a linear gradient began at 2 min to 25.4% B at 7 min, 90% at 7.2 min, 90% at 9 min, 20% at 9.1 min and 20% at 14 min. A 4000 QTRAP mass spectrometer coupled to the same LC column and time program as used in HPLC was used to collect all compound mass and fragmentation data. For fragmentation data and program setting details see Table S4. For norbelladine kinetics an MRM program in positive ion mode was used to monitor the following fragments 260.00 [M+H]^+^/138.00 [M-C_8_H_9_O]^+•^ *m/z*, 260.00 [M+H]^+^/121.00 [M-C_7_H_8_NO_2_]^+•^ *m/z*, 274.00 [M+H]^+^/137.00 [M+H-C_8_H_9_O_2_]^+^ *m/z*, 274.00 [M+H]^+^/122.00 [M+H-C_8_H_10_NO_2_]^+^ *m/z*. The fragments with 260.00 [M+H]^+^ *m/z* and 274.00 [M+H]^+^ *m/z* molecular ions were replaced when looking at *N*-methylnorbelladine for 274.00 [M+H]^+^/152.10 [M-C_8_H_9_O]^+•^ *m/z*, 274.00 [M+H]^+^/121.00 [M-C_9_H_12_NO_2_]^+•^ *m/z*, 288.00 [M+H]^+^/150.10 [M-C_8_H_9_O_2_]^+•^ *m/z* and 288.20 [M+H]^+^/137.00 [M-C_9_H_12_NO]^+•^ *m/z*. Papaverine internal standard was monitored with the following fragments 340.40[M+H]^+^/324.20 [M-CH_3_]^+•^ *m/z* and 340.40 [M+H]^+^/202.10 [M-C_8_H_9_O_2_]^+•^ *m/z.* When conducting dopamine kinetics, galanthamine was used as the internal standard and samples were not ethyl acetate extracted prior to LC/MS/MS analysis. To remove protein, two volumes of acetonitrile were added followed by 1 hr at −20°C and 10 min centrifugation at 16,100×g, 4°C. The supernatant was dried under vacuum and re-suspended in the starting mobile phase before analysis. The HPLC time program was changed to start at 5% solvent B with solution going to waste until 3.9 min, at 5 min start linear gradient to 25% B at 25 min, 90% B at 9.5 min, 90% B at 11 min, 5% B at 11.1 min and 5% B at 16 min. Ions monitored in the MRM were 168.00 [M+H]^+^/151.00 [M+H-OH]^+^ *m/z* and 168.00 [M+H]^+^/119.00 [M-OH-OCH_3_]^+•^ *m/z*. AdoMet steady state kinetic parameters were determined with norbelladine as the saturated substrate. Product was quantitated using HPLC with the 28 min program used for screening enzyme assays. Product for assays on the additional *Np*N4OMT variants was detected with this same 28 min program on HPLC.

When conducting kinetic experiments the *K_m_* was at least five fold higher than the minimum concentration of substrate and fivefold lower than the maximum concentration of substrate tested. *K_m_* and *k_cat_* were calculated by nonlinear regression to the Michaelis-Menten kinetics equation with the GraphPad PRISM 5.0 software.

### NMR

NMR spectra were acquired for 4′-*O*-methylnorbelladine in CD_3_OD at 600 MHz on a BrukerAvance 600 MHz spectrometer equipped with a BrukerBioSpin TCI 1.7 mm MicroCryoProbe. Proton, gCOSY, ROESY, gHSQC, and gHMBC spectra were acquired; ^13^C chemical shifts were obtained from the HSQC and HMBC spectra. Chemical shifts are reported with respect to the residual non-deuterated MeOD signal (δ_H_ 3.31) (Figure S1, S2, S3, S4 and S5).

### Quantitative Real Time-PCR (qRT-PCR)

cDNA for leaf, bulb and inflorescence tissues of daffodil were created using 1 µg RNA from the respective tissues, random primers and M-MLV reverse transcriptase according to the Invitrogen protocol. qRT-PCR was conducted with a TaqMan designed gene expression assay for the methyltransferase with ribosomal RNA as a reference according to manufacture protocol. Reactions (5 µl) were performed in quadruplicate with outlier exclusion using Applied Biosystems StepOnePlus Real-Time PCR system. Methyltransferase relative expression values were determined by calculating ΔΔC_T_ values relative to standard ribosomal RNA and leaf tissue [42].

## Results

**Figure 2:**
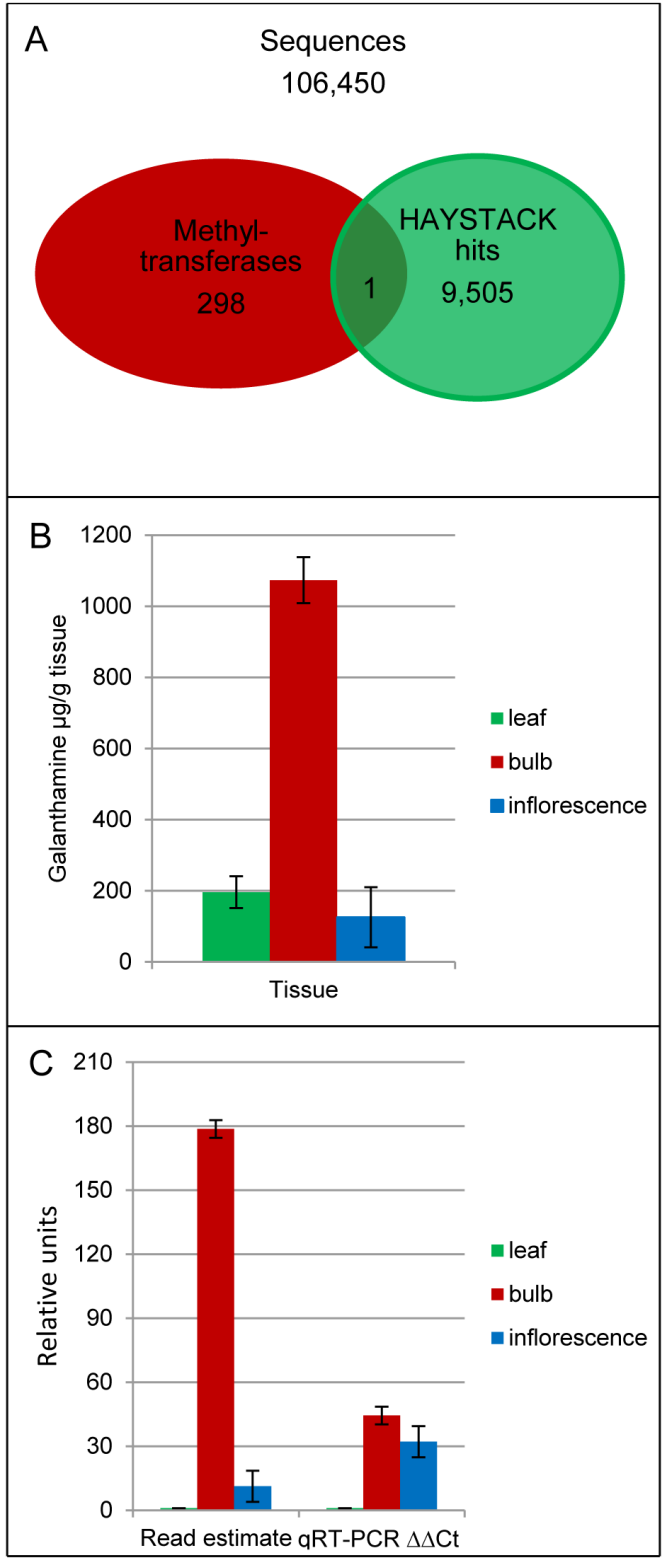
Identification of *Np*N4OMT in the daffodil transcriptome. (A) Venn diagram of all sequences, all OMTs and all galanthamine correlating sequences according to HAYSTACK. (B) Accumulation level of galanthamine in daffodil (C) Candidate *Np*N4OMT expression profile in leaf, bulb and inflorescence with the relative initial read estimate and qRT-PCR ΔΔCt on the y-axis with leaf tissue set to 1.

The Illumina sequencing of daffodil leaf, bulb and inflorescence tissues resulted in 65 million paired-end reads that were used to make the daffodil transcriptome assembly. The transcriptome assembly consisted of 106,450 sequences (Figure 2A) with a mean length of 551 base pairs and a maximum length of 13,381 base pairs. A similar number of >100 base pair sequences were found in the transcriptome of *Chlorophytum borivilianum* [43]. This mean length indicates a high number of the sequences are long enough for homology searches and cloning work. Of these sequences, 79,980 were predicted to have open reading frames and were translated into peptides. After determining the reads coming from the three tissues, several homologs of genes with predictable expression patterns were used to evaluate the quality of the expression estimations. The RuBisCO large and small subunits have high amounts of expression in the photosynthetic leaf and inflorescence tissues compared to the non-photosynthetic bulb tissue. A homolog to the MADS62 floral development transcription factor is exclusively expressed in the inflorescence tissue as would be expected [44]. The read counts were thus determined to produce expected expression patterns.

The LC/MS/MS data for leaf, bulb, and inflorescence tissues resulted in a pronounced accumulation pattern of galanthamine. The largest concentration was found in bulb tissue, with a lower level found in leaf and the lowest level in inflorescence (Figure 2B).

**Figure 3:**
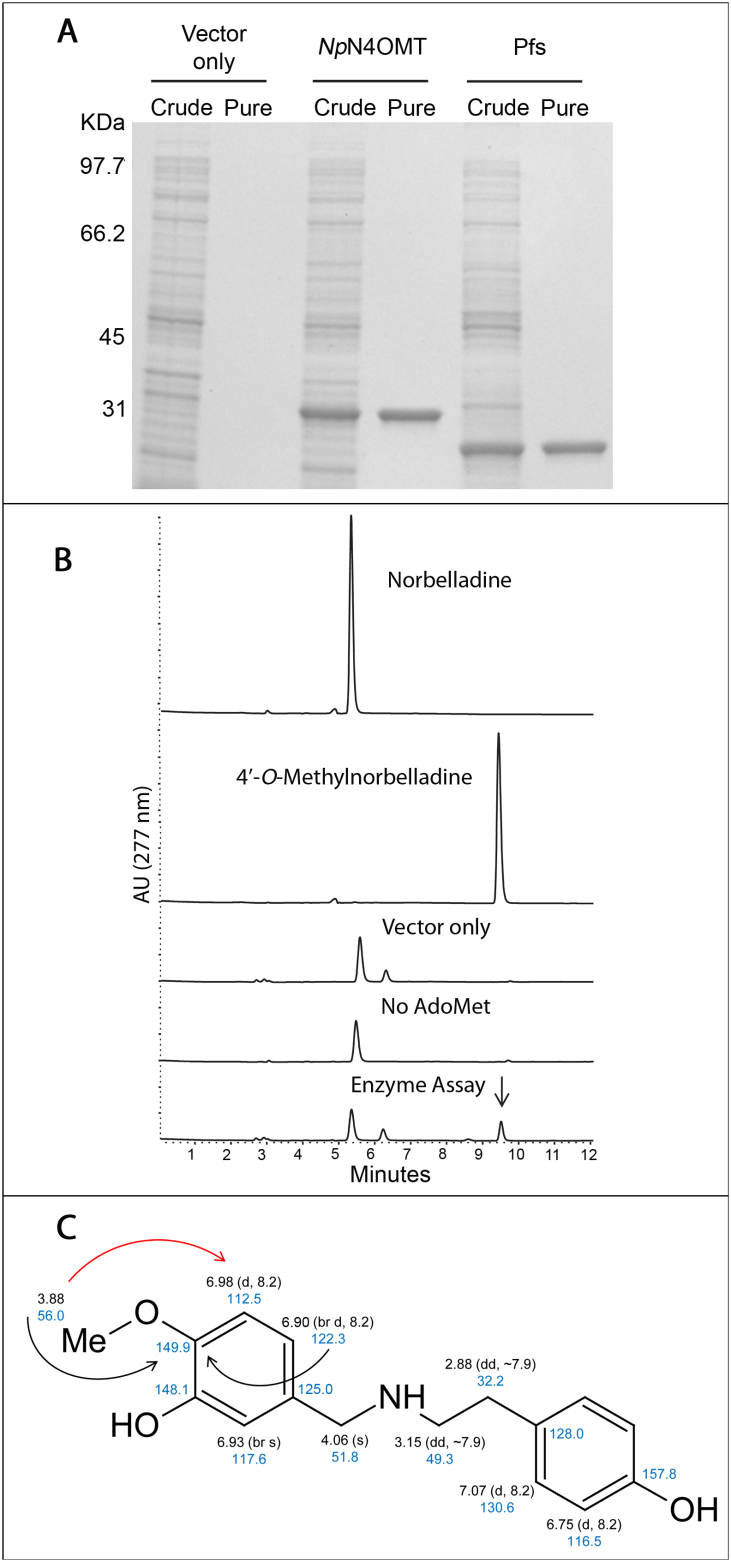
Recombinant *Np*N4OMT1 purification, enzyme assay and NMR structure elucidation of the 4′-*O-*methylnorbelladine product. (A) SDS-PAGE gel 10% including fractions from crude extract and the desalted protein prep. This is shown for vector only, *Np*N4OMT1 and Pfs preparations. (B) Enzyme assays (top to bottom): Norbelladine standard; 4′-*O-*Methylnorbelladine standard; Assay with *E. coli* vector-only crude extract added; Assay without AdoMet added; Complete methyltransferase assay. (C) NMR structure elucidation: proton chemical shifts are black, carbon chemical shifts are blue, key HMBC correlations are black arrows, and key ROESY correlations are red arrows.

Using BLAST to seek homologs to the methyltransferases found in Table S1 yielded 298 methyltransferase candidate genes [21]. Separately, HAYSTACK identified 9,505 contigs that co-express with galanthamine accumulation. A comparison of the two resulting lists revealed one methyltransferase *Np*N4OMT that fits the HAYSTACK model (Figure 2A). This methyltransferase was chosen for functional analysis. After RACE, *Np*N4OMT was found to be a 239 amino acid protein with a predicted molecular weight (MW) of 27 kDa. When expressed using the pET28a vector, the added *N*-terminal Histidine tag increased the MW to 29 kDa (Figure 3A). In the course of cloning, five unique clones were obtained with >96% identity to each other. Due to the two-toned yellow flower color, single flower and size, the daffodil variety used in this study is likely Carlton. Based on genome size estimates, Carlton is thought to be a domesticated form of *Narcissus pseudonarcissus* with a genome duplication event that resulted in a tetraploid [45]. A high number of paralogs is, therefore, expected. In addition, these bulbs have been propagated vegetatively. For these reasons, the existence of multiple similar sequences is not surprising.

Due to the high similarity of the clones, the first to be cloned was selected for thorough characterization. The clone selected for characterization is 92.5% identical on the amino acid level to the original sequence in the transcriptome assembly (Figure S6). The recombinant protein was purified with a yield of 16.7 mg protein/L *E. coli* culture. SDS-PAGE analysis revealed the protein to be of apparent homogeneity (Figure 3A). Initial enzyme assays with *Np*N4OMT1 yielded, upon HPLC analysis, a peak with the retention time of 4′-*O*-methylnorbelladine. The vector only control lacks *Np*N4OMT1 but has all other assay components. Therefor the absence of product in the vector control assay excludes the possibility of a background reaction. The absence of product in the assay lacking AdoMet shows that the methyltransferase uses AdoMet as a co-substrate and cannot form product without AdoMet (Figure 3B). The pH optimum was found to be 8.8 and the temperature optimum 45°C (Figure S7B–C). The Pfs protein, shown purified in Figure 3A, was added to prevent SAH inhibition in kinetic enzyme assays, through the Pfs catalyzed hydrolysis of SAH to adenine and *S*-ribosyl-homocysteine [41].

An alternative methylation product, 3′-*O-*methylnorbelladine, has the same retention time on HPLC, the same UV profile and MS/MS fragmentation pattern as 4′-*O*-methylnorbelladine. Thus, NMR analysis was performed to determine the regiospecificity of *O*-methylation. HMBC correlations from both the methoxyl protons (δ_H_ 3.88) and H-6′ (δ_H_ 6.90) to the same carbon (δ_C_ 149.9) placed the methoxyl group at C-4′. Its location was further supported by a ROESY correlation from the methoxyl protons to H-5′ (δ_H_ 6.98). The NMR data thus confirmed that 4′-*O*-methylnorbelladine is the product of the enzyme reaction (Figure 3C).

To determine the substrate specificity of this methyltransferase, several similar substrates were tested. Activity comparable to that found with norbelladine was observed using *N*-methylnorbelladine as the substrate. Dopamine also served as a substrate. Under the assay conditions used, product was not detected with caffeic acid, vanillin, 3,4-dihydroxybenzaldehyde, and tyramine as substrates (Table 1). To determine if the other 4 variants show similar activity, they were purified, and enzymatic activity was confirmed for all variants using norbelladine as the substrate. When monitoring *Np*N4OMT1 norbelladine assays allowed to proceed to completion, no sign of double methylation products were observed as expected.

**Table 1:** Substrate specificity of *Np*N4OMT1.

Substrate	*K*_m_ (µM)	*k*_cat_ (1/min)	*k*_cat_/*K*_m_ (1/µM*min)
norbelladine	1.6±0.3	1.3±0.06	0.8
AdoMet	28.5±1.6	4.5±0.01	0.16
*N-*methylnorbelladine	1.9±0.4	2.6±0.15	1.3
dopamine	7.3±2.7	3.6±0.15	0.5
caffeic acid	ND	ND	ND
vanillin	ND	ND	ND
3,4-dihydroxybenzaldehyde	ND	ND	ND
tyramine	ND	ND	ND

ND, Not detected.

±, Standard error.

**Figure 4:**
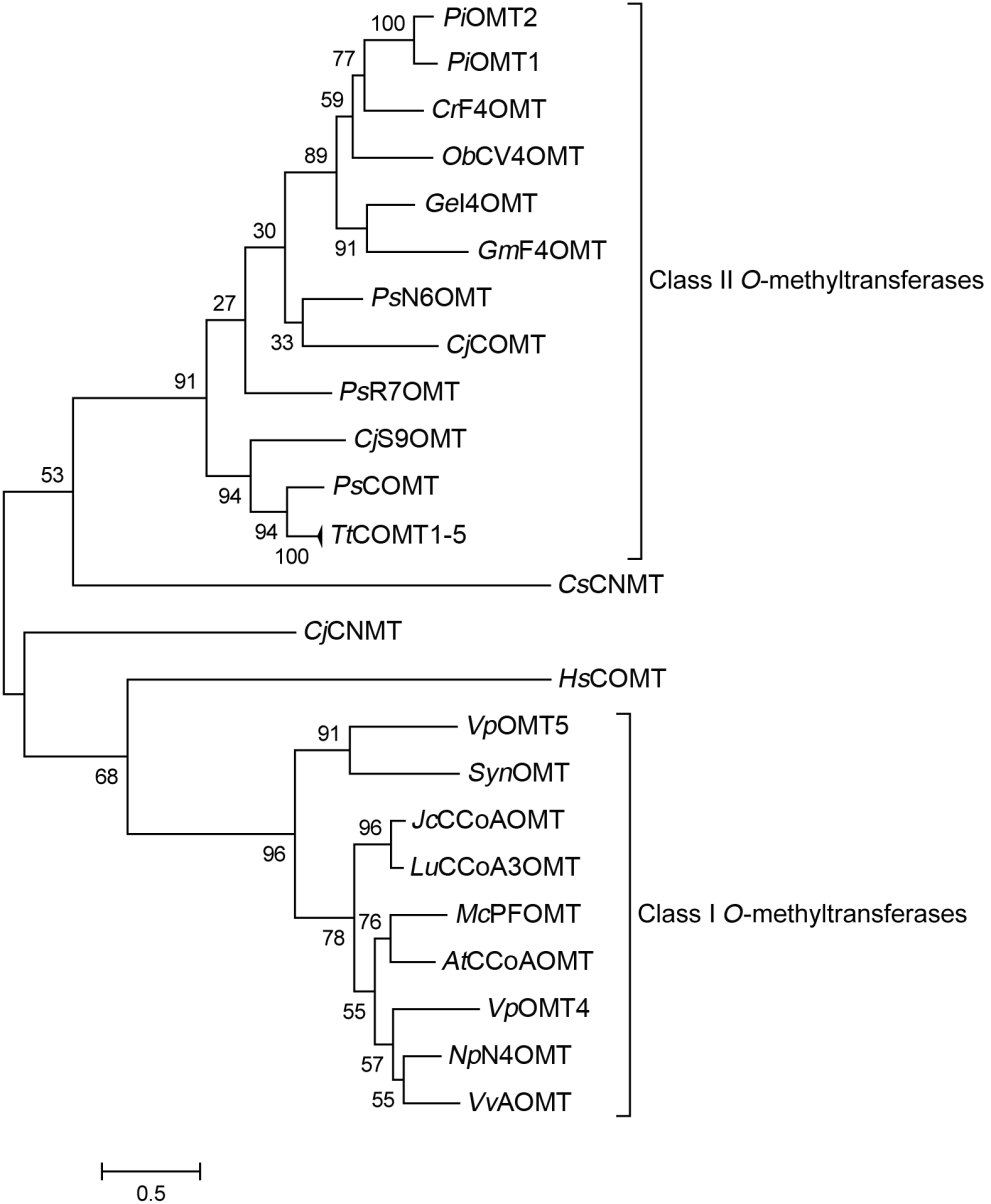
Phylogenetic analysis of *Np*N4OMT1. A maximum-likelihood phylogenetic tree of characterized methyltransferases listed in Table S2 [22]–[27], [46]–[49], [53], [63]–[70]. Alignment constructed using MUSCLE.

Phylogenetic analysis of the *Np*N4OMT1 placed it in the class I OMT group (Figure 4). *Np*N4OMT1 has a length consistent with the 231–248 amino acid range found in class I OMTs. This is in contrast to other known plant catechol 4-OMTs which all group in the class II OMTs as their length and cofactor requirements reported in previous work would predict. All these methyltransferases are significantly longer than the standard class I OMTs and none are reported to have the characteristic divalent cation dependence of class I OMTs [46]–[50]. When testing *Np*N4OMT1 for cation dependence, enzymatic activity improved upon the addition of cobalt. Enzymatic activity increased four-fold more with the addition of magnesium instead of cobalt (Figure S7A). This preference for magnesium over other divalent cations is also to be expected from a class I OMT [50]. It is, furthermore, consistent with previous work on enzyme extracts enriched for this OMT [9].

To validate the expression profiles predicted based on read counts; qRT-PCR was conducted with the same RNA preparation used to prepare the cDNA libraries for Illumina sequencing. The resulting expression profile is slightly different from that obtained from Illumina sequencing. The qRT-PCR expression profile has a higher quantity of inflorescence transcript relative to bulb transcript (Figure 2C). This minor difference is potentially due to cross amplification, during qRT-PCR, with other close homologs in the plant.

## Discussion

The expression pattern, product formation and low *K*_m_ for norbelladine all indicate *Np*N4OMT methylates norbelladine in the proposed galanthamine biosynthetic pathway. Two differing orders of methylation have been proposed for galanthamine biosynthesis [10]. The methylation of *N*-methylnorbelladine was tested to determine if a preference for the *N*-methylation state could be observed at *O*-methylation. Similar *K_m_* and *k_cat_* values for *N*-methylnorbelladine and norbelladine indicate that a preference for the *N*-methylation state does not occur at *O*-methylation. The results presented here support both proposed galanthamine biosynthetic pathways. Future work on additional enzymes in the pathway will be needed to enzymatically validate one pathway or the other. The lack of enzymatic activity when testing 3,4-dihydroxybenzaldehyde suggests that methylation does not occur prior to formation of norbelladine. The methylation of dopamine is expected considering structural similarity to the methylated moiety of norbelladine. Tyramine was not methylated; this is as expected for a class I OMT (Table 1).

Several aspects of the candidate gene selection approach proved important for this successful identification. One is the selection of a variety of methyltransferases for the homology search. If only the known 4-OMTs had been used in the homology search, the gene would have been missed due to the large difference in sequence between known 4-OMTs and *Np*N4OMT. It has been shown that catechol 4′-OMT and catechol 3′-OMT can differ by as little as one amino acid [51]. Because of this potential for a conversion form catechol 3′-*O*-methylation to 4′-*O*-methylation though evolution, OMTs of both positions were used in the homology search. Also, both class I and class II OMTs were used in the search because both classes are known to methylate catechols. Considering the multiple branches of the *N*-methyltransferases in the OMT phylogeny, it is worth investigating enzymes that annotate as *N*-methyltransferases [21]. For these reasons, the sequences used in the initial BLAST search consisted of representatives of known *O-* and *N*-methyltransferases of small metabolites. The *Np*N4OMT turned out to be a member of the class I OMTs. Class I OMTs show closer homology to human catechol OMT than to all known plant catechol 4-OMTs in class II, as demonstrated in Figure 4 [52]. The closest known catechol 4-OMT to *Np*N4OMT is bacterial, has 34% identity to *Np*N4OMT and is a class I OMT from *Cyanobacterium Synechocystis* sp. Strain PCC 6803 (*Syn*OMT) [53]. Many 3-OMTs show even higher homology to *Np*N4OMT than *Syn*OMT. It is probable that the 4-OMT activity of *Np*N4OMT was acquired independently of *Syn*OMT (Figure 4).

The second selection criterion, co-expression with galanthamine accumulation, was also of great value. It reduced the number of candidate OMTs from hundreds to one. There are a variety of methods for the prioritization of candidate genes [54], [55]. Many of these methods are oriented towards species and systems for which there are extensive databases or prior knowledge regarding a gene involved in the pathway or process. In one study, a collection of ∼500 microarrays was used to demonstrate the co-expression of genes in the same pathway in Arabidopsis [56]. However, extensive gene expression data are typically not available for non-model systems. There have been several studies that use co-expression analysis to find genes in a pathway and produce promising candidate gene lists. These studies often lack biochemical validation of the in *silico* candidates [57]. If a novel function is proposed, this type of analysis is incomplete without biochemical validation of enzyme activity. Enzymes that are homologous to functionally equivalent enzymes in a different species can be corroborated by co-expression analysis [57]. There are several studies that use a simple differential expression model and microarrays to find biosynthetic genes by comparing biosynthetically active and inactive accessions in rose and strawberry [58], [59]. Differential expression analysis lacks algorithms to use data with differing levels of metabolism occurring in more than two samples. The Pearson correlation can compare data from multiple samples. Mercke et al. have used a Pearson correlation-based method to identify gene expression with microarrays that correlate with levels of specific terpenes in cucumber [60]. Illumina-based transcriptomes are, however, more sensitive to minor variants in the sequences and to splice variants. Illumina-based gene expression data also have a far greater dynamic range, limited only by sequence depth, than microarrays [61]. Subtleties in the sequences that could be missed with microarrays can now be detected with Illumina sequencing.

The use of HAYSTACK as a platform to use the Pearson correlation is ideal because it is designed to receive a hypothesis for gene expression and look for gene expression that correlates with that hypothesis. This is in contrast to an approach in which gene expression patterns are clustered based on similarity to each other. The search for a defined pattern in the data allows the number of required expression data points to be reduced compared to an approach that needs to define clusters of gene expression patterns based on similarity. In HAYSTACK, the shared expression pattern is already defined. HAYSTACK applies additional screening criteria including a p-value test for significance, a fold cutoff and background cutoff. The approach chosen in our study used knowledge of known chemical intermediates, a transcriptome with expression profiles for three tissues, and metabolite levels to identify a candidate gene product to validate with *in vitro* enzyme activity. Little prior knowledge of a pathway is required to use this approach, making this workflow ideal for the identification of genes in unknown biochemical pathways.

There are several modifications to this approach that could be used to improve its power. It could be applied to more tissues, environmental conditions or time points to provide even greater statistical power to correlate co-expression of biosynthetic genes with the biosynthesis of their products. The method could also be modified to include analysis of multiple end products. If the pathway in which the enzyme participates branches, several end products could be equally important to co-expression analysis. This combined consideration of multiple end products could lead to more informative models [62]. Another potential source of information on the metabolite level could be the concentrations of intermediates made during synthesis. Correlations between biosynthetic gene expression, and perhaps the accumulation of metabolites as well, tend to decrease as distance in a pathway increases [56]. Experiments that quantitate metabolic intermediates could be useful for finding biosynthetic genes if the flux through the pathway is not so high that intermediates do not accumulate. The latter would be the case in a pathway assembled into a metabolon.

The discovery of this enzyme enables the future elucidation of other enzymes in the proposed galanthamine biosynthetic pathway and other novel pathways. Genes that co-express with *Np*N4OMT can be identified and used as candidate genes for other steps in the proposed galanthamine biosynthetic pathway. This will potentially be useful for earlier steps in the pathway, considering the tendency of expression correlations to decrease as distance in metabolic pathways increase [56]. This enzyme discovery also validates the utility of this workflow to characterize metabolic pathways and provides a valuable method for pathway discovery in orphan species.

## Supporting Information

Figure S1 ***Np*** **N4OMT1 product 4′-** ***O*** **-methylnorbelladine proton NMR spectra with peak assignments.**
(TIF)

Figure S2 ***Np*** **N4OMT1 product 4′-** ***O*** **-methylnorbelladine COSY spectra.**
(TIF)

Figure S3 ***Np*** **N4OMT1 product 4′-** ***O*** **-methylnorbelladine HMBC spectra.**
(TIF)

Figure S4 ***Np*** **N4OMT1 product 4′-** ***O*** **-methylnorbelladine ROESY spectra.**
(TIF)

Figure S5 ***Np*** **N4OMT1 product 4′-** ***O*** **-methylnorbelladine HSQC spectra.**
(TIF)

Figure S6 **Protein sequence alignment of** ***Np*** **N4OMT variants.** Five unique variants of the *Np*N4OMT sequence are aligned against the original sequence predicted by the *de novo* assembled transcriptome using CLC software. Dots are identical residues.
(TIF)

Figure S7 **Effect of divalent cations, temperature and pH on** ***Np*** **N4OMT1 enzyme activity.** (A) Divalent cations tested with 5 min assays with 5 µM of cation Ca^2+^, Co^2+^, Zn^2+^, Mg^2+^ or Mn^2+^. (B) pH optimum 15 min assays with 5 µM Mg^2+^. (C) Temperature optimum 15 min assays with 5 µM Mg^2+^. Divalent cation and pH testing reactions are 100 µl reactions at 37°C. The divalent cation test contained 4 µM norbelladine, while pH and temperature optimum tests contained 100 µM norbelladine in the assay mix.
(TIF)

Table S1 **Methyltransferases used in BLAST search.**
(DOCX)

Table S2 **Methyltransferases used in phylogeny.**
(DOCX)

Table S3 **Primers used in RACE, cloning and colony PCR.**
(DOCX)

Table S4 **Parameters used for LC/MS/MS analysis.**
(DOCX)
