# Supplementary figures and images for: Cloning and Characterization of a Norbelladine 4′-O-Methyltransferase Involved in the Biosynthesis of the Alzheimer’s Drug Galanthamine in Narcissus sp. aff. pseudonarcissus
Source: PLoS One. 2014 Jul 25;9(7):e103223. doi: 10.1371/journal.pone.0103223 (PMC4111509; doi:10.1371/journal.pone.0103223)

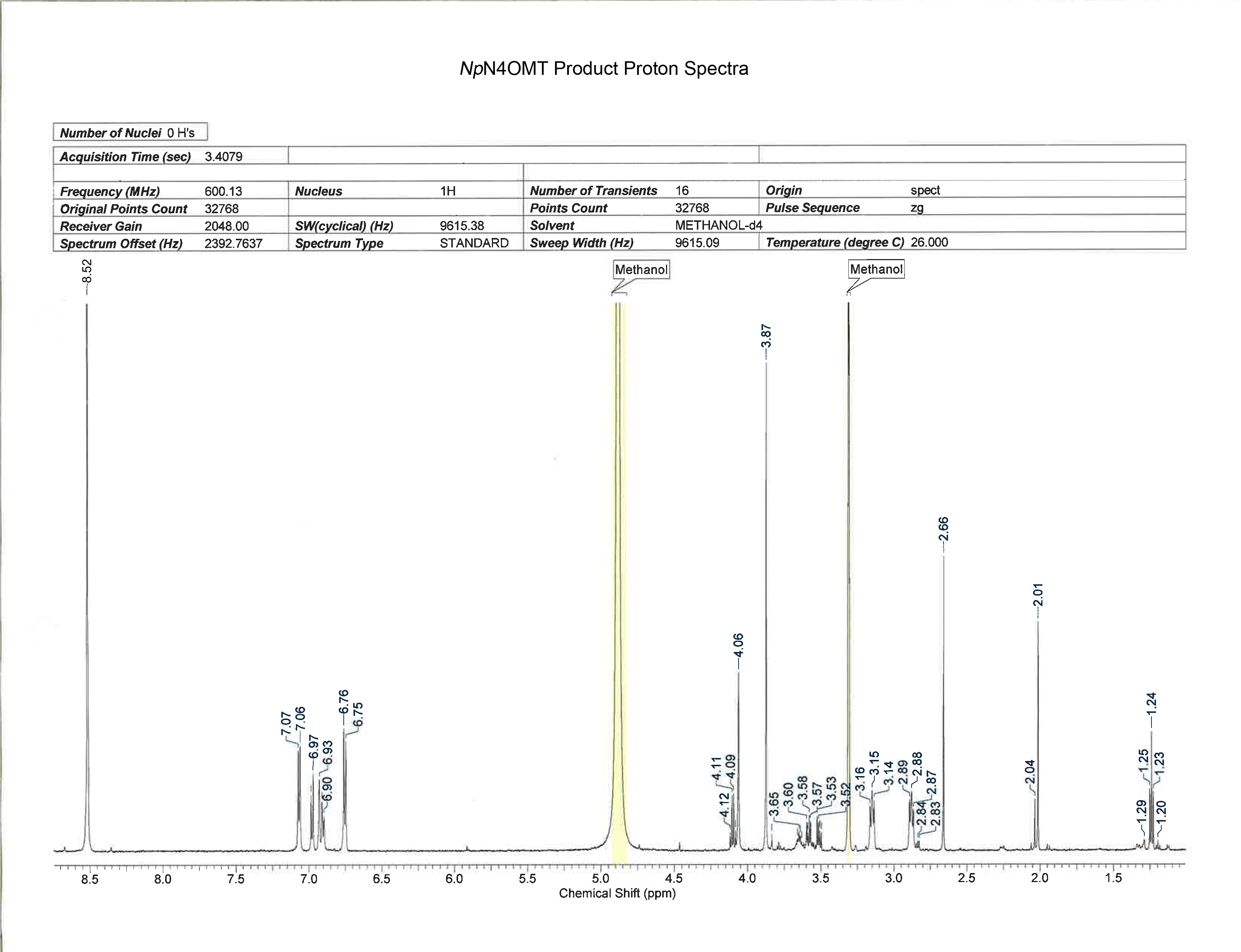

Supplement: Figure S1 — Np N4OMT1 product 4′- O -methylnorbelladine proton NMR spectra with peak assignments. (TIF) [file pone.0103223.s001.tif]

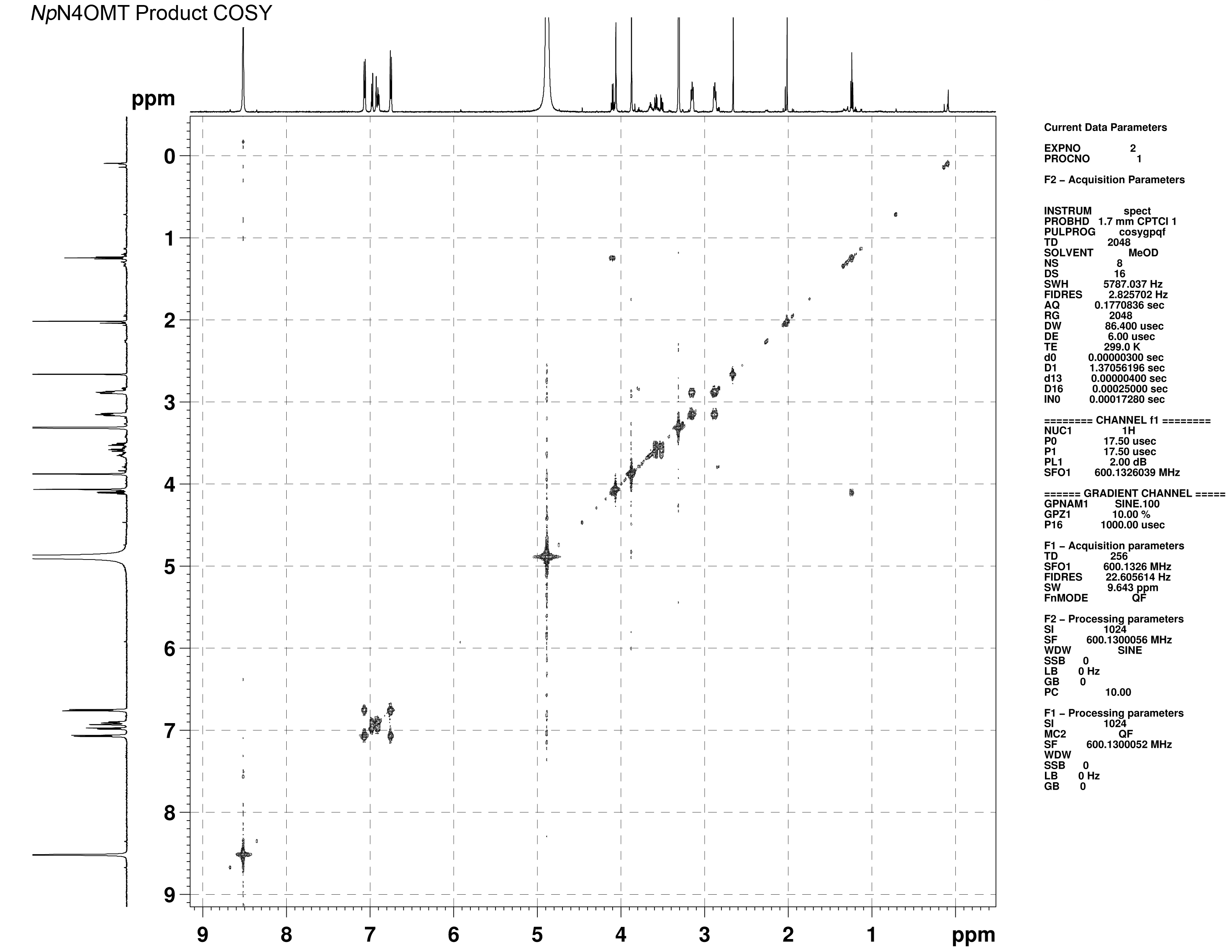

Supplement: Figure S2 — Np N4OMT1 product 4′- O -methylnorbelladine COSY spectra. (TIF) [file pone.0103223.s002.tif]

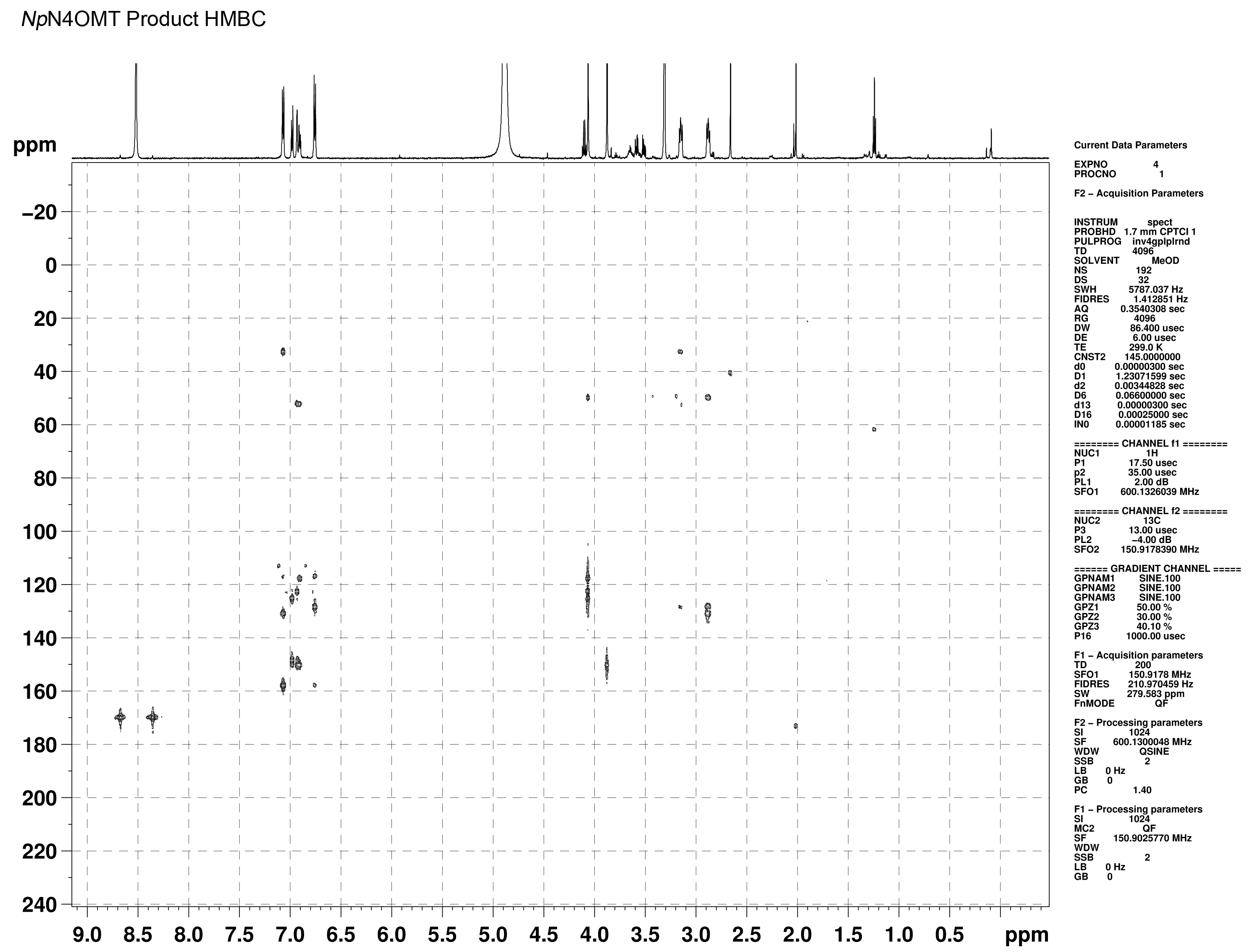

Supplement: Figure S3 — Np N4OMT1 product 4′- O -methylnorbelladine HMBC spectra. (TIF) [file pone.0103223.s003.tif]

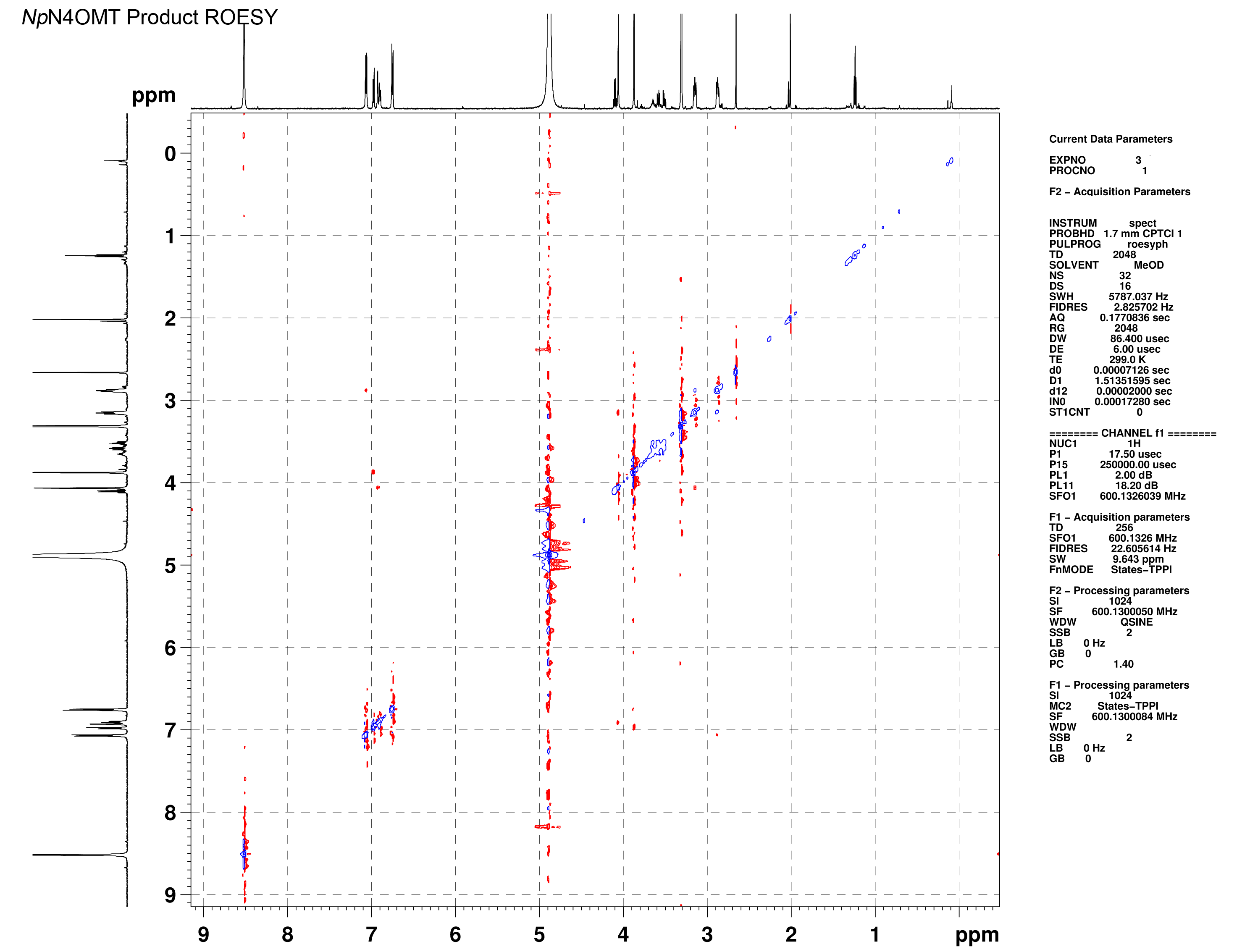

Supplement: Figure S4 — Np N4OMT1 product 4′- O -methylnorbelladine ROESY spectra. (TIF) [file pone.0103223.s004.tif]

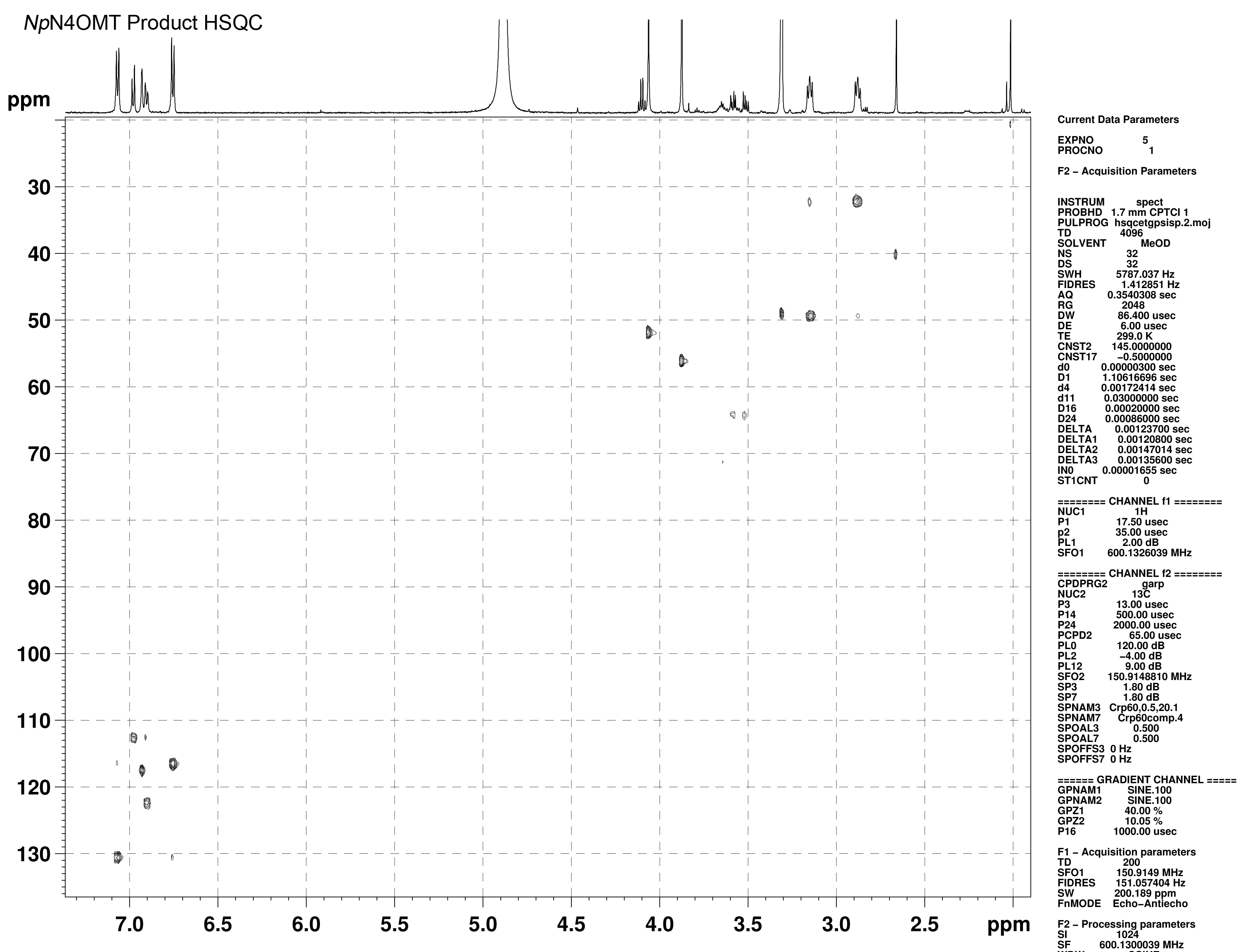

Supplement: Figure S5 — Np N4OMT1 product 4′- O -methylnorbelladine HSQC spectra. (TIF) [file pone.0103223.s005.tif]

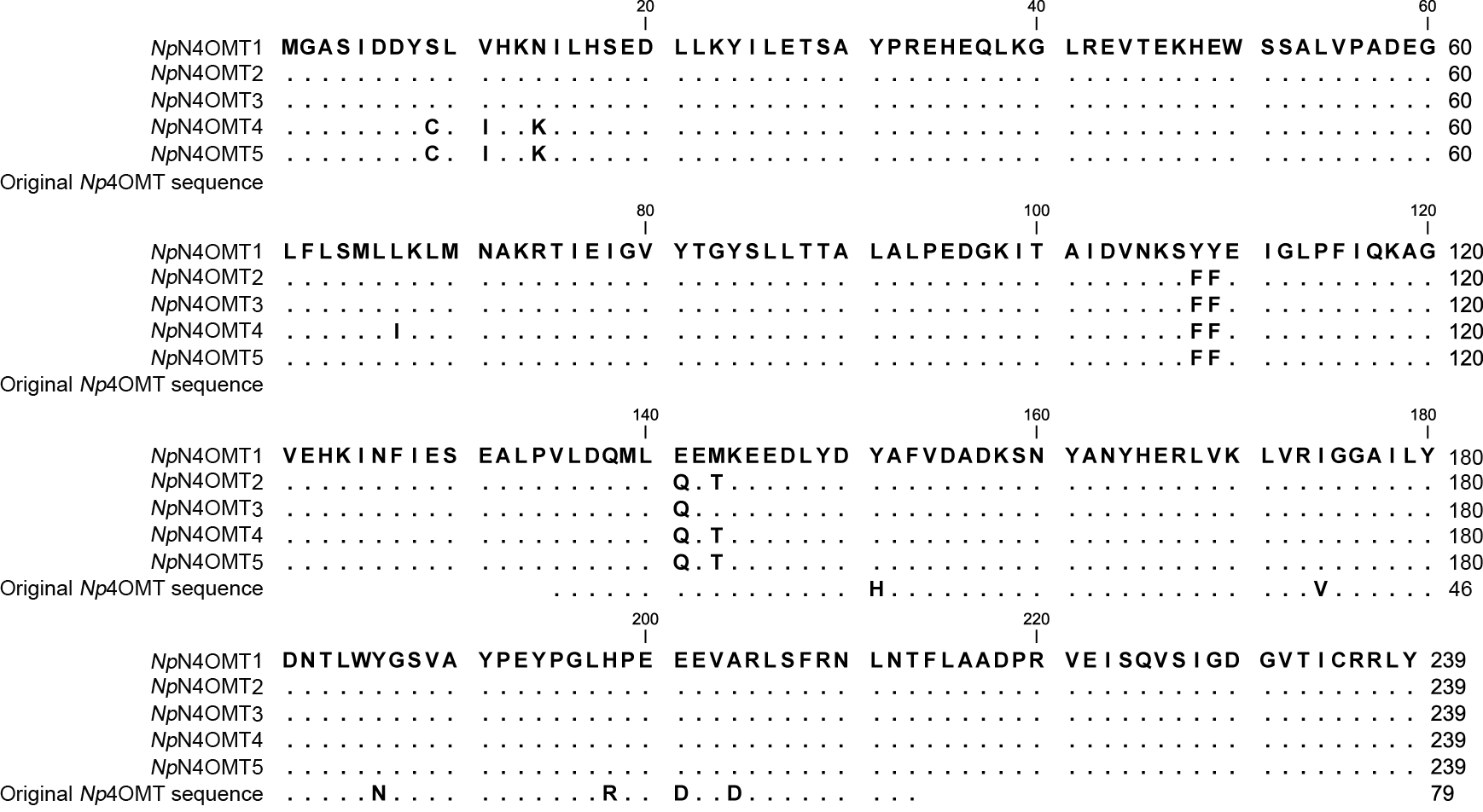

Supplement: Figure S6 — Protein sequence alignment of Np N4OMT variants. Five unique variants of the NpN4OMT sequence are aligned against the original sequence predicted by the de novo assembled transcriptome using CLC software. Dots are identical residues. (TIF) [file pone.0103223.s006.tif]

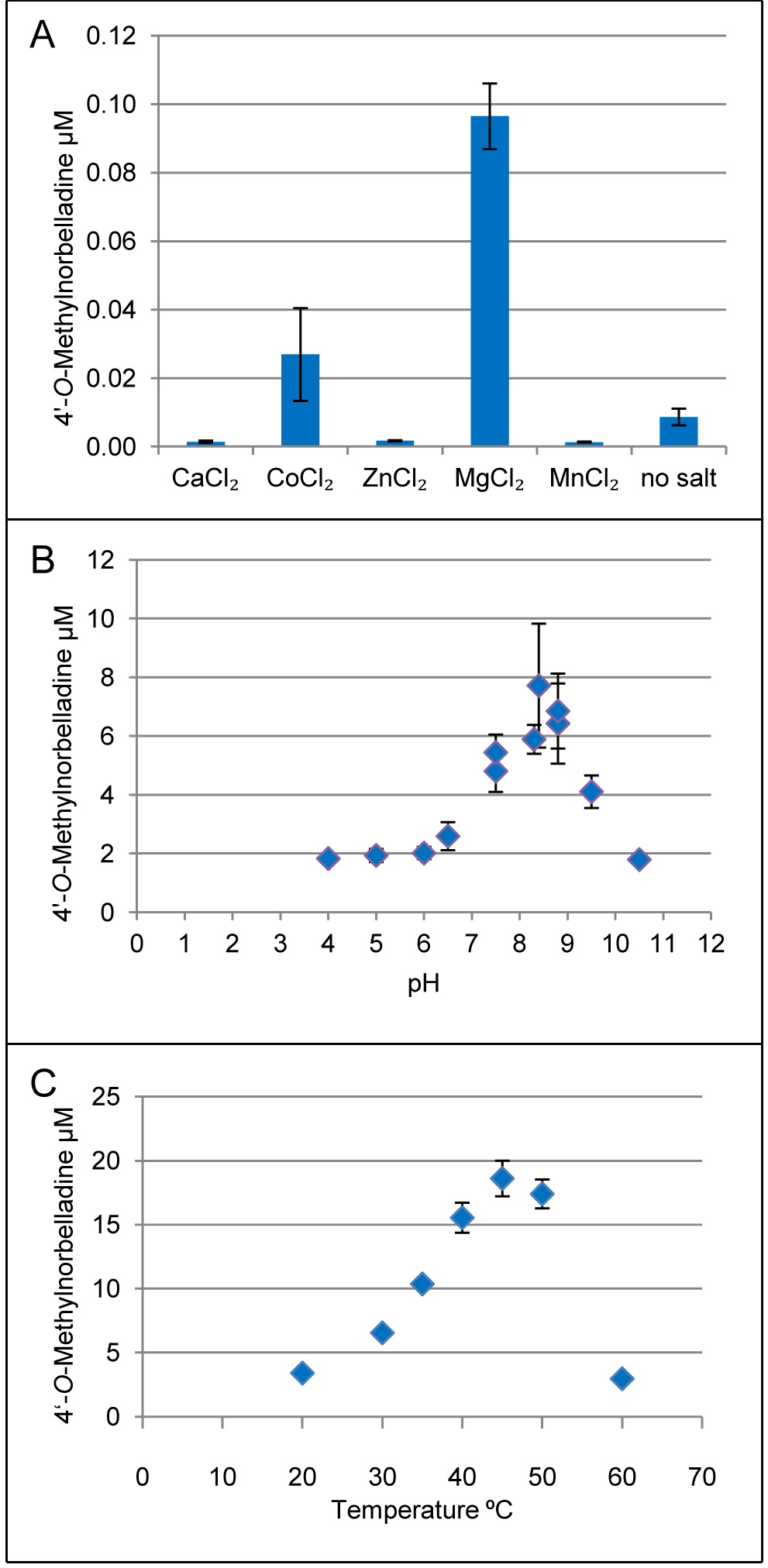

Supplement: Figure S7 — Effect of divalent cations, temperature and pH on Np N4OMT1 enzyme activity. (A) Divalent cations tested with 5 min assays with 5 µM of cation Ca2+, Co2+, Zn2+, Mg2+ or Mn2+. (B) pH optimum 15 min assays with 5 µM Mg2+. (C) Temperature optimum 15 min assays with 5 µM Mg2+. Divalent cation and pH testing reactions are 100 µl reactions at 37°C. The divalent cation test contained 4 µM norbelladine, while pH and temperature optimum tests contained 100 µM norbelladine in the assay mix. (TIF) [file pone.0103223.s007.tif]
