# Supplementary material for: Cloning and Characterization of a Norbelladine 4′-O-Methyltransferase Involved in the Biosynthesis of the Alzheimer’s Drug Galanthamine in Narcissus sp. aff. pseudonarcissus
Source: PLoS One. 2014 Jul 25;9(7):e103223. doi: 10.1371/journal.pone.0103223 (PMC4111509; doi:10.1371/journal.pone.0103223)
Supplement: Table S1 — Methyltransferases used in BLAST search. (DOCX) [file pone.0103223.s008.docx]

**Table S1**. Methyltransferases used in BLAST search

| Accession number | Substrate specificity | Reference |
| --- | --- | --- |
| AAQ01669.1 | (*R*,*S*)-norcoclaurine, (*R*)-norprotosinomenine, (*S*)-norprotosinomenine, (*R*,*S*)-isoorientaline, | [23] |
| AAQ01670.1 |  | unpublished |
| AAQ01668.1 | guaiacol, isovanillic acid, (*R*)-reticuline, (*S*)-reticuline, (*R*,*S*)-orientaline, (*R*)-protosinomenine, (*R*,*S*)-laudanidine | [23] |
| BAI79244.1 | (1*S*)-*N*-deacetylisoipecoside, (1*R*)-*N*-deacetylipecoside, (13a*R*)-demethylalangiside, (11bS)-7′-*O*-demethylcephaeline, (13a*S*)-redipecamine, (1*R*,*S*)-isococlaurine, (1*R*,*S*)-norcoclaurine, (1*R*,*S*)-isoorientaline, oripavine | [24] |
| BAI79245.1 | (13a*S*)-3-*O*-methylredipecamine, (1*S*)-coclaurine, (1*R*,*S*)-*N*-methylcoclaurine, (1*R*,*S*)-4′-*O*-methylcoclaurine, (1*R*,*S*)-6-O-methyllaudanosoline, (1*R*,*S*)-nororientaline, (1*S*)-norreticuline, (1*S*)-reticuline, (13a*S*)-coreximine | [24] |
| BAI79243.1 | (1*S*)-*N*-deacetylisoipecoside, (1*S*)-7-*O*-methyl-*N*-deacetylisoipecoside, (11b*S*)-cephaeline, (1*R*,*S*)-isococlaurine, (1*R*,*S*)-norcoclaurine, (1*S*)-4′-*O*-methyllaudanosoline, (1*R*,*S*)-nororientaline, (1*R*,*S*)-isoorientaline, (1*S*)-norprotosinomenine, (1*R*)-norprotosinomenine, (1*R*,*S*)-protosinomenine | [24] |
| BAA06192.1 | (*R*,*S*)-scoulerine | [22] |
| AAD29843.1 | see reference | [26] |
| AAD29841.1 | see reference | [26] |
| AAD29845.1 | see reference | [26] |
| AAD29842.1 | see reference | [26] |
| AAD29844.1 | see reference | [26] |
| BAC22084.1 | columbamine,  tetrahydrocolumbamine, (*S*)-scoulerine, 2,3,9,10-tetrahydroxyprotoberberine | [25] |
| ACV50428.1 | homology with caffeoyl-CoA *O-*methyltransferase described in [63] | [64] |
| AAN61072.1 | quercetin, 7-*O*-methylquercetin, quercetin-3-*O*-glucoside, quercetagetin, 3-*O*-methylquercetagetin 6-*O*-methylquercetagetin, 6-hydroxykaempferol, myricetin, luteolin, caffeoyl-CoA | [52] |
| AAR02420.1 | eriodictyol, homoeriodictyol, kaempferol, quercetin, isorhamnetin, chrysoeriol | [46] |
