# Supplementary material for: Cloning and Characterization of a Norbelladine 4′-O-Methyltransferase Involved in the Biosynthesis of the Alzheimer’s Drug Galanthamine in Narcissus sp. aff. pseudonarcissus
Source: PLoS One. 2014 Jul 25;9(7):e103223. doi: 10.1371/journal.pone.0103223 (PMC4111509; doi:10.1371/journal.pone.0103223)
Supplement: Table S2 — Methyltransferases used in phylogeny. (DOCX) [file pone.0103223.s009.docx]

**Table S2.** Methyltransferases used in phylogeny

| Accession number | Short name | Species | Substrate specificity | Reference |
| --- | --- | --- | --- | --- |
| AAQ01669.1 | *Ps*N6OMT | *Papaver somniferum* | (*R*,*S*)-norcoclaurine, (*R*)-norprotosinomenine, (*S*)-norprotosinomenine, (*R*,*S*)-isoorientaline, | [23] |
| AAQ01670.1 | *Ps*COMT | *Papaver somniferum* |  | unpublished |
| AAQ01668.1 | *Ps*R7OMT | *Papaver somniferum* | guaiacol, isovanillic acid, (*R*)-reticuline, (*S*)-reticuline, (*R*,*S*)-orientaline, (*R*)-protosinomenine, (*R*,*S*)-laudanidine | [23] |
| BAI79244.1 | *Pi*OMT2 | *Psychotria ipecacuanha* | (1*S*)-*N*-deacetylisoipecoside, (1*R*)-*N*-deacetylipecoside, (13a*R*)-demethylalangiside, (11b*S*)-7′-*O*-demethylcephaeline, (13a*S*)-redipecamine, (1*R*,*S*)-isococlaurine, (1*R*,*S*)-norcoclaurine, (1*R*,*S*)-isoorientaline, oripavine | [24] |
| BAI79243.1 | *Pi*OMT1 | *Psychotria ipecacuanha* | (1*S*)-*N*-deacetylisoipecoside, (1*S*)-7-*O*-methyl-*N*-deacetylisoipecoside, (11b*S*)-cephaeline, (1*R*,*S*)-Isococlaurine, (1*R*,*S*)-norcoclaurine, (1*S*) 4′*O*-methyllaudanosoline, (1*R*,*S*)-nororientaline, (1*R*,*S*)-isoorientaline, (1*S*)-norprotosinomenine, (1*R*)-norprotosinomenine, (1*R*,*S*)-protosinomenine | [24] |
| BAA06192.1 | *Cj*S9OMT | *Coptis japonica* | (*R*,*S*)-scoulerine | [22] |
| AAD29843.1 | *Tt*COMT3 | *Thalictrum tuberosum* | see reference | [26] |
| AAD29841.1 | *Tt*COMT1 | *Thalictrum tuberosum* | see reference | [26] |
| AAD29845.1 | *Tt*COMT5 | *Thalictrum tuberosum* | see reference | [26] |
| AAD29842.1 | *Tt*COMT2 | *Thalictrum tuberosum* | see reference | [26] |
| AAD29844.1 | *Tt*COMT4 | *Thalictrum tuberosum* | see reference | [26] |
| BAC22084.1 | *Cj*COMT | *Coptis japonica* | columbamine,  tetrahydrocolumbamine, (*S*)-scoulerine, 2,3,9,10-tetrahydroxyprotoberberine | [25] |
| ACV50428.1 | *Jc*CCoAOMT | *Jatropha curcas* | homology with caffeoyl-CoA *O-*methyltransferase described in [63] | [64] |
| AAR02420.1 | *Cr*F4OMT | *Catharanthus roseus* | eriodictyol, homoeriodictyol, kaempferol, quercetin, isorhamnetin, chrysoeriol | [46] |
| Q9C5D7.1 | *At*CCoAOMT | *Arabidopsis thaliana* | not determined | [65] |
| C7AE94.1 | *Vv*AOMT | *Vitis vinifera* | cyanidin 3-glucoside, delphinidin 3-glucoside, quercetin 3-glucoside, cyanidin, quercetin, myricetin, pelargonidin 3-glucoside, catechin, epicatechin | [66] |
| ADZ76153.1 | *Vp*OMT4 | *Vanilla planifolia* | tricetin, 5-hydroxyferulic acid ethyl ester, 5-hydroxyferulic acid, myricetin, 3,4-dihydroxybenzaldehyde, quercetin, 5-hydroxyconiferaldehyde, caffeoyl CoA, caffeic acid ethyl ester, caffeoylaldehyde, caffeic acid | [27] |
| ADZ76154.1 | *Vp*OMT5 | *Vanilla planifolia* | tricetin, 5-hydroxyferulic acid ethyl ester, 5-hydroxyferulic acid, myricetin, 3,4-dihydroxybenzaldehyde, quercetin, 5-hydroxyconiferaldehyde, caffeoyl CoA, caffeic acid ethyl ester, caffeoylaldehyde, caffeic acid | [27] |
| Q84KK6 | *Ge*I4OMT | *Glycyrrhiza echinata* | 2,7,4'-trihydroxyisoflavanone, medicarpin | [47] |
| C6TAY1 | *Gm*F4OMT | *Glycine max* | apigenin, daidzein, genistein, quercetin, naringenin | [48] |
| AAY89237.1 | *Lu*CCoA3OMT | *Linum usitatissimum* |  | [67] |
| 3C3Y\|A | *Mc*PFOMT | *Mesembryanthemum crystallinum* | quercetin, quercetagetin, caffeic acid, CoA, caffeoyl glucose | [68] |
| 62361_DF6 | *Np*N4OMT1 | *Narcissus* sp. *aff. pseudonarcissus* | norbelladine, *N-*methylnorbelladine, dopamine | this study |
| BAB71802.1 | *Cj*CNMT | *Coptis japonica* | (*R*)-coclaurine, (*S*)-coclaurine, (*R*,*S*)-norreticuline, (*R*,*S*)-norlaudanosoline, (*R*,*S*)-6-*O-*methylnorlaudanosoline, 6,7-dimethoxyl-1,2,3,4-tetrahydroisoquinoline, 1-methyl-6,7-dihydroxy-1,2,3,4-tetrahydroisoquinolinne | [69] |
| BAB12278.1 | *Cs*CNMT | *Camellia sinensis* | 7-methylxanthine, 3-methylxanthine, 1-methylxanthine, theobromine, theophylline, paraxanthine | [70] |
| Q93WU3 | *Ob*CV4OMT | *Ocimum basilicum* | chavicol, phenol, eugenol, t-isoeugenol, t-anol | [49] |
| Q8WZ04 | *Hs*COMT | *Homo sapiens* | a catechol |  |
| 3CBG\|A | *SynOMT* | *Cyanobacterium*  *Synechocystis*  sp. strain PCC 6803 | hydroxyferulic acid, caffeic acid, caffeoyl-CoA, caffeoylglucose, 3,4,5-trihydorxycinnamic acid, tricetin, 3,4-dihydroxybenzoic acid | [53] |
