# Supplementary material for: Cloning and Characterization of a Norbelladine 4′-O-Methyltransferase Involved in the Biosynthesis of the Alzheimer’s Drug Galanthamine in Narcissus sp. aff. pseudonarcissus
Source: PLoS One. 2014 Jul 25;9(7):e103223. doi: 10.1371/journal.pone.0103223 (PMC4111509; doi:10.1371/journal.pone.0103223)
Supplement: Table S3 — Primers used in RACE, cloning and colony PCR. (DOCX) [file pone.0103223.s010.docx]

**Table S3.** Primers used in RACE, cloning and colony PCR

| Primer name | Primer sequence |
| --- | --- |
| 62361_5’_RACE_outer | TCCACCTCATCTTCCGGACGAA |
| 62361_5’_RACE_inner | ACTTCCGTTCCAGAGCGTGTT |
| 62361_3’_RACE_outer | AGAAGACCTGTACGACCATGCAT |
| 62361_3’_RACE_inner | ACGAGCGATTAGTGAAGCTCGTCA |
| 62361_forward_outer | CTTCACTTGTGTCAAGTTCAAT |
| 62361_reverse_outer | CCRATAGATAGCATGCAGAATCT |
| 62361_forward_inner | ^a,b^aattCATATGGGTGCTAGCATAGATGATT |
| 62361_reverse_inner | ^a,b^aattGCGGCCGCTCAATAAAGACGTCGGCAAATAGT |
| qRT-PCR_forward_62361 | ATTGGTGTGTACACCGGCTATT |
| qRT-PCR_reverse_62361 | TTCCATCTTCCGGTAAAGCCAAA |
| qRT-PCR_probe_62361 | CTCTGCTCACAACTGC |
| T7 sequencing | TAATACGACTCACTATA |
| T7 terminator | GCTAGTTATTGCTCAGCGG |

^a^Lower case bases are extra for restriction enzyme binding.

^b^Underlined bases are added restriction sites.
