# Supplementary material for: Cloning and Characterization of a Norbelladine 4′-O-Methyltransferase Involved in the Biosynthesis of the Alzheimer’s Drug Galanthamine in Narcissus sp. aff. pseudonarcissus
Source: PLoS One. 2014 Jul 25;9(7):e103223. doi: 10.1371/journal.pone.0103223 (PMC4111509; doi:10.1371/journal.pone.0103223)
Supplement: Table S4 — Parameters used for LC/MS/MS analysis. (DOCX) [file pone.0103223.s011.docx]

**Table S4.** Parameters used for LC/MS/MS analysis

| Compound | Predicted molecular ion *m/z* [M+H] | Fragments *m/z* (% relative intensity)[proposed fragment] | CE value (V) | DP value (V) | Injection volume (µl) |
| --- | --- | --- | --- | --- | --- |
| galanthamine | 288.14 |  | 35 | 70 | 10 |
| norbelladine* | 260.13 | 121.04(100.00)[M-OH-C_8_H_9_O]^+●^, 121.84(19.62), 122.00(13.29)[M+H-C_7_H_8_O]^+^, 122.64(10.13), 123.04(38.61)[M-C_8_H_10_ON]^+●^, 123.68(11.39), 138.00(3.16)[M-C_8_H_9_O]^+●^, 260.16(21.52)[M+H]^+^ | 15 | 60 | 10 |
| 4’-*O*-methylnorbelladine* | 274.14 | 122.08(1.63)[M+H-C_8_H_10_O_2_N]^+^, 137.04(100.00) [M-C_8_H_10_ON]^+●^**,** 274.08(2.45)[M+H]^+^ | 35 | 60 | 10 |
| *N*-methylnorbelladine | 274.14 | 121.04(100.00)[M-C_8_H_10_O_2_N]^+●^**,** 121.52(19.11), 122.00(18.18), 123.04(82.29)[M-C_9_H_12_ON]^+●^, 123.68(17.69), 124.00(16.43), 124.56(15.03), 124.96(10.53), 152.16(73.72)[M-C_8_H_9_O]^+●^, 274.08(28.54)[M+H]^+^, | 20 | 60 | 10 |
| 4’-*O*-methyl-*N*-methylnorbelladine* | 288.18 | 137.04(100.00)[M-C_9_H_12_ON]^+●^, 150.08(1.22)[M-C_8_H_9_O]^+●^, 288.08(18.67)[M+H]^+^ | 20 | 60 | 10 |
| dopamine* | 154.09 | 91.04(41.26), 119.04(24.85)[M-OH-OH]^+●^, 137.04(100.00)[M+H-OH]^+^, 137.92(10.21), 154.08(1.29)[M+H]^+^ | 20 | 70 | 20 |
| 3’-*O-*methyldopamine | 168.10 | 90.96(47.83), 91.60(10.87)[M-OH-CH_3_-C_2_H_6_N]^+●^, 94.88(11.87), 95.20(10.87), 118.72(15.22), 119.04(39.13)[M-OH-OCH_3_]^+●^, 140.20(13.04)[M-CH-CH_3_]^+●^, 152.40(10.87)[M+H-NH_2_]^+^, 151.04(100.00)[M+H-OH]^+^, 151.60(13.04), 168.16(52.17)[M+H]^+^, | 20 | 70 | 20 |
| methylated dopamine product | 168.10 | 91.04(41.18)[M-OH-CH_3_-C_2_H_6_N]^+●^, 92.08(11.76)[M+H-OH-CH_3_-C_2_H_6_N]^+^, 109.28(11.76)[M+H-CH_3_-C_2_H_6_N]^+^, 112.08(17.65), 119.04(29.41)[M-OH-OCH_3_]^+●^, 123.00(17.65)[M-C_2_H_6_N]^+●^, 126.00(11.76), 136.00(17.65)[M+H-OH-CH_3_]^+^, 150.56(17.65)[M-OH]^+●^, 151.04(100.00)[M+H-OH]^+^, 151.60(17.65), 154.32(17.65), 168.08(94.12)[M+H]^+^, 168.48(17.65), 169.68(11.76), | 20 | 70 | 20 |
| papaverine | 340.16 | 171.12(47.37)[M-C_8_H_9_O_2_-OCH_3_]^+●^, 172.08(11.94)[M+H- C_8_H_9_O_2_-OCH_3_]^+^, 187.04(11.23)[M-C_8_H_9_O_2_-CH_3_]^+●^, 202.08(48.17)[M-C_8_H_9_O_2_]^+●^, 280.08(17.59)[M+H-N-CH_3_-OCH_3_]^+^, 296.08(16.81)[M+H-N-CH_3_-CH_3_]^+^, 308.08(25.35)[M-OCH_3_]^+●^, 324.08(100.00)[M-CH_3_]^+●^, 340.08(1.16)[M+H]^+^ | 52 | 70 | 10 |

*Cut off for inclusion in fragments is 10 % relative intensity. If parent ions or fragments used in MRM are below this threshold, these ions are reported.
